# Influence of the Growth Parameters on RF-Sputtered CNTs and Their Temperature-Selective Application in Gas Sensors

**DOI:** 10.1021/acsomega.5c03699

**Published:** 2025-08-04

**Authors:** Mikayel Aleksanyan, Artak Sayunts, Gevorg Shahkhatuni, Zarine Simonyan, Davit Kananov, Rima Papovyan, Dušan Kopecký

**Affiliations:** † Center of Semiconductor Devices and Nanotechnologies, 105430 Yerevan State University, 1 Alex Manoogian, 0025 Yerevan, Armenia; ‡ Department of Mathematics, Informatics and Cybernetics, Faculty of Chemical Engineering, 52735 University of Chemistry and Technology Prague, Technická 5, Prague 6, 166 28 Prague, Czech Republic

## Abstract

This work deals with the peculiarities of the growth of carbon nanotubes (CNTs) by radiofrequency (RF) magnetron sputtering and with the effect of deposition parameters on the RF sputtering. In the deposition process, a type of plasma gas, power of the RF generator, deposition time of catalysts, and a type of catalyst metals were modified to reveal the impact of these changes on the CNT’s growth. The obtained nanostructures were studied by scanning electron microscopy (SEM) and transmission electron microscopy (TEM) as well as energy-dispersive X-ray (EDX) and Raman spectroscopies. The best results were obtained when the deposition conditions were as follows: argon-assisted plasma, generator power 120 W, catalyst sputtering duration 20 s, and nickel serving as a catalyst. A flexible propylene glycol vapor (PGV) and hydrogen peroxide vapor (HPV) sensors based on RF-sputtered CNTs combined with the Fe_2_O_3_:ZnO material were fabricated, and its DC and AC gas-sensing properties were studied. Impedance spectroscopy was used to evaluate an equivalent electrical circuit of the sensor. Temperature modulation led to the effective use of the same nanostructured film for PGV and HPV detection at 150 and 50 °C, respectively. At 50 °C temperature, the sensor response ranged from 3 to 27 values in the HPV concentrations of 0.5–25 ppm, respectively, demonstrating short response/recovery times, high response repeatability, and temporal stability.

## Graphical abstract

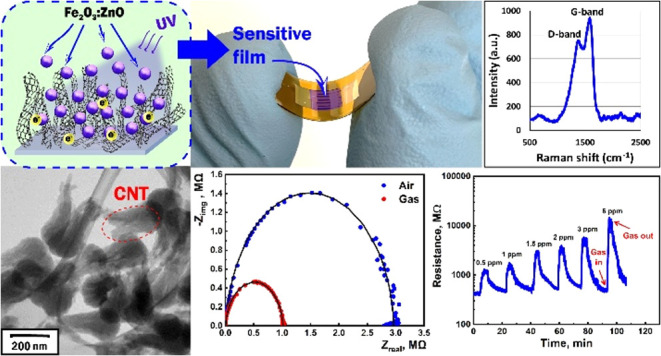

## Introduction

Carbon nanotubes (CNTs) have been at the center of attention since their discovery due to their unique physical, chemical, optical, and mechanical properties.^1–3^ Along with the development of various synthesis routes, it is possible to prepare CNTs with different morphologies, sizes, and perfection, such as single-walled CNTs, multiwalled CNTs, Y-shaped CNTs, zigzag-shaped CNTs, C60/C70, carbon nanowires, and bamboo-like CNTs.^4,5^ Such a variety of CNTs are advantageous in different branches of contemporary nanotechnology research. CNTs are characterized by incredible mechanical strength, high charge carrier mobility, chemical stability, flexibility (electron mobility: (10^4^–10^5^) cm^2^/(V × s), tensile strength: ∼100 GPa, Young’s modulus: ∼1 to 1.28 TPa, thermal conductivity: 0.1–6600 Wm^–1^ K^–1^, density: 1.3–1.4 g/cm^3^, specific strength: 48,000 kNm/kg, electrical conductivity: 10^6^ S m^–1^ (SWCNT (single-walled carbon nanotubes)) and 10^5^ S m^–1^ (MWCNT (multiwalled carbon nanotubes)), Raman: G band = ∼1584 cm^–1^, G band = ∼2600 to 2800 cm^–1^, D band = ∼1300 to 1400 cm^–1^),^6,7^ and compatibility with modern micro/nanoelectronic systems.^8–10^ They are widely used in sensor systems on the research and development level, both as piezoelectric cells and gas-sensitive layers.^11–14^ In particular, their large effective surface area and high charge carrier mobility create incomparably good conditions for their application as a gas-sensitive matrix.^15^

The technological evolution of the last decades has improved methods for the preparation of nanostructures, making it possible to control almost all parameters of final supramolecular systems. A general objective is to prepare CNTs with a perfect hexagonal structure, which requires complex technological methods and advanced skills.^16,17^ The most common methods of CNT preparation are arc discharge and laser ablation, belonging to the PVD (physical vapor deposition) series. The growth of CNTs by PVD methods is quite sophisticated and easy to apply.^18^ The use of the chemical vapor deposition (CVD) method to grow CNTs is also widespread, and improving its attributes is an ongoing process.^19–21^ Even though the CVD method is quite popular and has a number of advantages, the radiofrequency (RF) magnetron sputtering method can be considered as a suitable and equivalent alternative.^22–24^ High-frequency magnetron sputtering is an advanced deposition method that allows for the production of nanostructures with controllable parameters. It is technologically easy to use and requires low maintenance (argon gas, water cooling, etc.).^25^ In this method, the dynamic process of nanotube growth is largely controlled by the generator power, process duration, target-substrate distance, and catalyst particle distribution, etc. Moreover, the free growth of nanotubes is greatly influenced by the type of plasma gas used, the density and type of catalyst particles, as well as the vacuum conditions.^26,27^

Propylene glycol has attracted considerable interest in industrial and domestic applications. It is introduced into various food formulations, and its presence in the fumes emitted from food is the best indicator of the freshness and purity level. It is also emitted as a result of the vaping of modern electronic cigarettes, the concentration of which can be used to assess the degree of danger of cigarettes to the human body. Thus, it is still a challenge to measure its vapor concentrations accurately.^28–30^

Detection of hydrogen peroxide vapor (HPV) in the environment is also currently challenging, mainly related to medical and pharmaceutical applications. It has excellent decontamination properties and is used as a disinfectant for various types of medical surfaces and wounds.^31^ Ideal decontamination of pharmaceutical production environments is mainly carried out using HPV, after which the problem arises of cleaning the environment itself from these vapors.^32^ Furthermore, monitoring the concentration of HPV in human exhaled air can serve as a noninvasive diagnostic mechanism for pulmonary diseases.^33^

Various composites based on CNTs and metal oxide nanoparticles (SnO_2_, ZnO, Fe_2_O_3_, In_2_O_3_, etc.) are particularly suitable for use in gas sensors,^34–38^ because the combination of properties of two or more chemical substances may result in higher sensor performance.^39–41^ We have previously synthesized and characterized CNTs using the RF magnetron sputtering.^39^ Recently, optimized CNTs have been developed by our research group, based on which Fe_2_O_3_:ZnO/CNTs chemoresistive flexible sensors for propylene glycol vapor (PGV) and HPV detection were also fabricated.^42^

This work aimed to identify possible factors affecting the growth parameters of CNTs by setting the main prerequisites and preconditions for their growth. Herein, the influence of a catalyst and plasma gas as well as sputtering duration and power were tested. The resulting nanostructures were studied using scanning electron microscopy (SEM), transmission electron microscopy (TEM), energy-dispersive X-ray (EDX), and Raman techniques. Moreover, the evaluation of gas-sensing characteristics of the Fe_2_O_3_:ZnO/CNTs sensor was also carried out by impedance spectroscopy at a deeper level. The selective detection of HPV at 50 °C was revealed by temperature modulation, demonstrating a response even at the parts per billion level (500 ppb). Thus, the novelty of the work was attributed to the discovery of the optimal combination of technological parameters for the growth of CNTs, in which case, the use of the resulting nanostructures as a gas-sensitive matrix led to the achievement of optimized gas-sensing parameters for HPV and PGV detection.

## Experimental Section

### Growth Parameters of CNTs

In our previous work,^42^ carbon nanotubes were successfully synthesized and characterized using the VTC-600-2HD DC/RF dual-head high vacuum magnetron plasma system. Here, the crystalline, compositional, and other properties of the grown CNTs were investigated in detail, unequivocally confirming the stable presence of CNTs on the silicon substrate. The entire process of the CNT growth was also discussed here, which is illustrated in Figure 1. This PVD-driven process uses plasma ions generated by RF magnetron from an inert gas to bombard a graphite target. Detached particles subsequently condense around the catalyst islands on the substrate. Nanoparticles of catalytic metals are predeposited onto the substrate by DC sputtering. As a result, nanotubes are formed according to the vapor/liquid/solid (VLS) or vapor–solid (VS) theories.^43–45^ In the case of magnetron sputtering, the VS theory was more likely to be valid, since the substrate temperature was much lower (<500 °C) during the growth process and reaching the liquid phase was less likely. Here, the main advantage was that the target particles (carbon), torn off by the impact of high-energy ions (nitrogen), also had high energy, which allowed their diffusion to the surface of the catalyst particles and the formation of nuclei. Besides, the nonoxidation process of the catalytic particles also created favorable conditions for the application of this theory and the free growth of nanotubes. The point was that, unlike other methods, here, the catalyst particles were deposited in the same vacuum process by DC sputtering without removing the oxygen environment. Their very short deposition time allowed for the obtaining of not a continuous metal layer but interrupted nano islands (nucleation sites) without using chemical etching methods. The nonoxidized surface of the catalytic (metallic) centers also facilitated the easy diffusion/absorption of incoming carbon particles, which greatly contributed to the easy growth of the nanotubes.^46–48^

**Figure 1:**
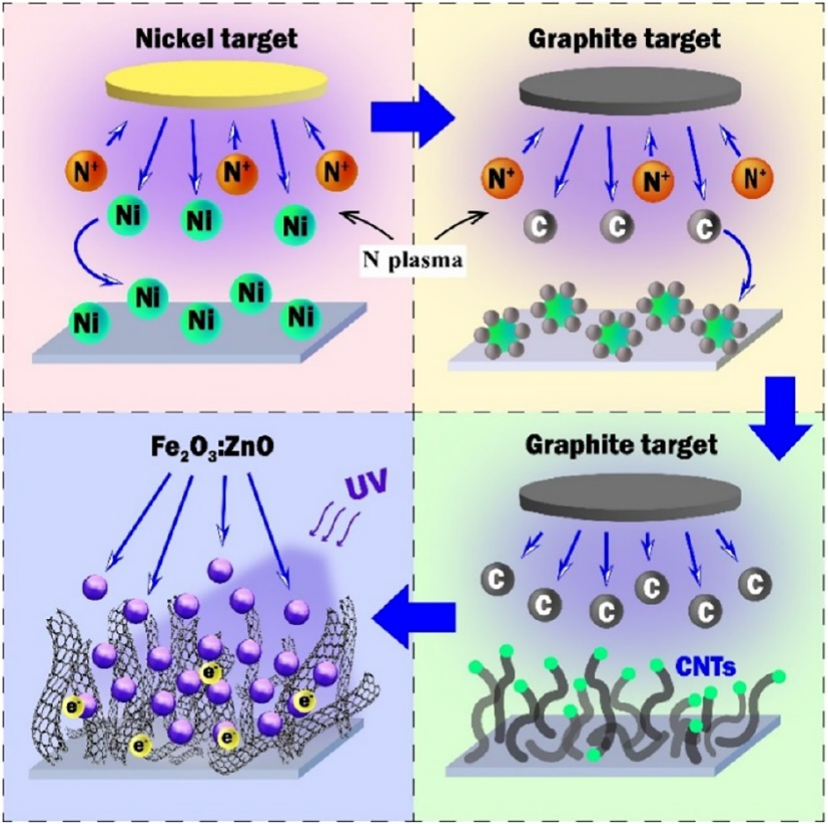
Process of the CNTs growing and subsequent preparation of the gas-sensing layer.

In this work, an experiment was set to investigate the effect of selected deposition parameters on nanotube growth. The high-purity targets (nickel, chrome, and graphite, 50 mm in diameter, N4 purity, Changsha Xinkang Advanced Materials Co., Ltd., China) and substrates (Si (100), Prime-Grade 2″ Silicon Wafers, N-Type, ρ = 10, α Nanotech Inc., Canada) were used. It is well-known that one of the main stimulating conditions for the growth of nanotubes in this method is the presence of a nitrogen-induced plasma.^24^ Therefore, nitrogen was used as the plasma gas, and the deposition time of nickel catalyst particles was set to 20 s, while the sputtering power from the graphite target was 120 W.

A plasma containing 100% pure argon and 100% pure oxygen were used for comparison under the same conditions in the nanotube growth process, confirming the indispensable role of nitrogen in the nanotube formation process. As a continuation of the research, we also increased the sputtering power from 120 to 140 W in the case of nitrogen plasma. This presumably led to the detachment of larger particles from the graphite target, which negatively affected the growth of the nanotubes. The main prerequisite for the growth of nanotubes was the presence of catalyst particles, the distribution and size of which are determined by the DC sputtering parameters and by the duration of the deposition process.^26^ Keeping other related parameters constant, we changed the sputtering duration for the nickel target from 10 to 30 s. The short sputtering times lead to the formation of sparsely distributed and smaller particles and vice versa. In the case of the longer deposition duration of catalyst particles, a certain transformation of nanotube growth was observed, while in the case of a shorter time, nanotubes were not formed at all.

The use of nickel catalyst has been proven many times, as it has excellent catalytic properties and is relatively inexpensive.^49,50^ However, the type of metal particles as catalysts also had a significant impact on the growth of nanotubes.^51,52^ For example, catalysts, such as chromium and iron, can also be successfully used in this process.^53–55^ Thus, keeping the basic parameters constant, nickel was replaced with chromium, which led to significant modifications of the formed nanostructures. All experimental regimes for growing nanostructures are summarized in Table 1, and the corresponding samples are labeled from SP1 to SP7.

**Table 1:** Experimental Regimes for Growing Nanostructures

sample no	process	sputtering duration	generator power	working pressure	sputtering gas	substrate temperature	cathode current	base pressure
SP1	Ni sputtering (DC)	20 s		4.8 × 10^–2^ Pa	nitrogen	400 °C	300 mA	8.3 × 10^–4^ Pa
	graphite sputtering (RF)	4 h	120 W	3.3 × 10^–2^ Pa	nitrogen	400 °C		8.1 × 10^–4^ Pa
SP2	Ni sputtering (DC)	20 s		4.7 × 10^–2^ Pa	argon	400 °C	300 mA	8.1 × 10^–4^ Pa
	graphite sputtering (RF)	4 h	120 W	3.1 × 10^–2^ Pa	argon	400 °C		8.2 × 10^–4^ Pa
SP3	Ni sputtering (DC)	20 s		4.6 × 10^–2^ Pa	oxygen	400 °C	300 mA	8.4 × 10^–4^ Pa
	graphite sputtering (RF)	4 h	120 W	3.3 × 10^–2^ Pa	oxygen	400 °C		8.3 × 10^–4^ Pa
SP4	Ni sputtering (DC)	20 s		4.8 × 10^–2^ Pa	nitrogen	400 °C	300 mA	8.53 × 10^–4^ Pa
	graphite sputtering (RF)	4 h	140 W	3.5 × 10^–2^ Pa	nitrogen	400 °C		8.1 × 10^–4^ Pa
SP5	Ni sputtering (DC)	10 s		4.4 × 10^–2^ Pa	nitrogen	400 °C	300 mA	8.6 × 10^–4^ Pa
	graphite sputtering (RF)	4 h	120 W	3 × 10^–2^ Pa	nitrogen	400 °C		8.2 × 10^–4^ Pa
SP6	Ni sputtering (DC)	30 s		4.7 × 10^–2^ Pa	nitrogen	400 °C	300 mA	8 × 10^–4^ Pa
	graphite sputtering (RF)	4 h	120 W	3.6 × 10^–2^ Pa	nitrogen	400 °C		8.4 × 10^–4^ Pa
SP7	Cr sputtering (DC)	20 s		4.1 × 10^–2^ Pa	nitrogen	400 °C	300 mA	7.9 × 10^–4^ Pa
	graphite sputtering (RF)	4 h	120 W	3.2 × 10^–2^ Pa	nitrogen	400 °C		8.1 × 10^–4^ Pa

It is often difficult to reach the same vacuum level in different experimental processes precisely, which is why slight changes in the vacuum levels were observed in the processes. Presumably, differences are negligible and did not affect the general physical phenomena or the outcome of the comparisons made.

Using the SP1 sample (CNTs), a PGV chemoresistive flexible sensor was produced by coating the surface of CNTs with the Fe_2_O_3_:ZnO nanoparticles (Figures 1 and 2).^42^ Gold interdigitated ohmic contacts were sputtered onto a polyimide flexible substrate by using a magnetron sputtering method. The CNTs and the Fe_2_O_3_:ZnO nanograins were deposited onto the contacts using the electron beam and the magnetron sputtering methods, respectively. The initial (baseline) resistance of the sensor was 100 MΩ at room temperature under normal atmospheric conditions. Besides, the SP1 sample (CNTs) was synthesized multiple times using the same fixed technological regimes, in which case, nanostructures with the same parameters were obtained, demonstrating the reproducibility of the production technology.

**Figure 2:**
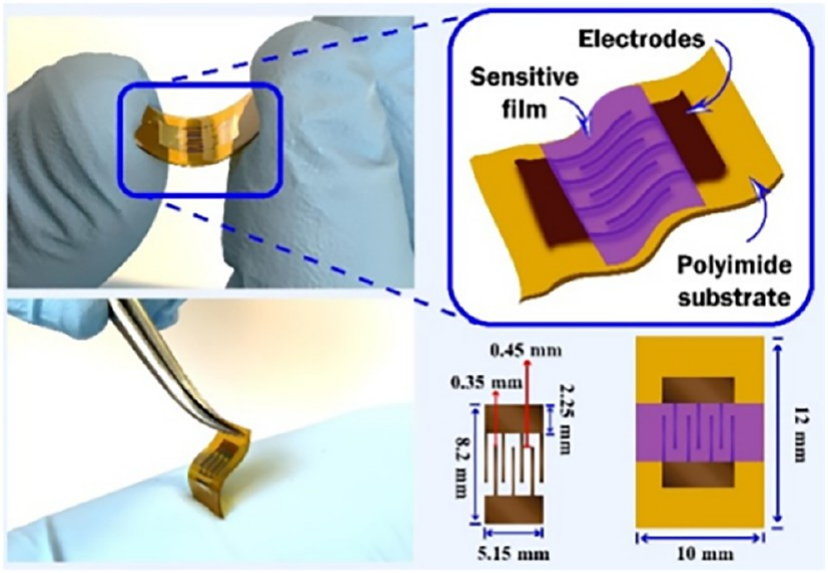
Actual photographs and schematic representation of the flexible Fe_2_O_3_:ZnO/CNTs sensor.

Subsequently, a laboratory-designed gas-sensing setup^56^ as well as the Electrochemical Workstation ZIVE SP1 and the Keithley 4200-SCS Parameter Analyzer techniques^57^ for impedance spectroscopy were used. The PGV response of the sensor was defined as the ratio of the sensitive film resistances in air (*R*_air_) and in the presence of PGV (*R*_gas_).

## Results and Discussion

### Characterization

The appearance and crystal structure of the synthesized and characterized CNTs (SP1, Table 1) are illustrated by SEM and TEM images (Figure 3a–c). The nanotubes are 150–200 nm in length and 40–80 nm in diameter. The Raman spectra of the CNTs revealed a typical hexagonal graphene structure indicated by D (1366 cm^–1^) and G (1582 cm^–1^) bands (Figure 3d).^42^ The value of the D/G ratio was higher (1.16), indicating higher defect level of CNTs.^58^ Besides, we had a rather higher *I*(D)/*I*(G) (1.24) ratio, which was also attributed to the availability of a higher number of structural defects with multiple graphite layers representing MWCNT structures.^59^ Besides, the SAED (selected area electron diffraction) pattern represented the nanocrystalline structure of the CNTs (Figure 3d), and the EDX elemental mapping analysis confirmed the homogeneous distribution of C and N elements (Figure 3f).

**Figure 3:**
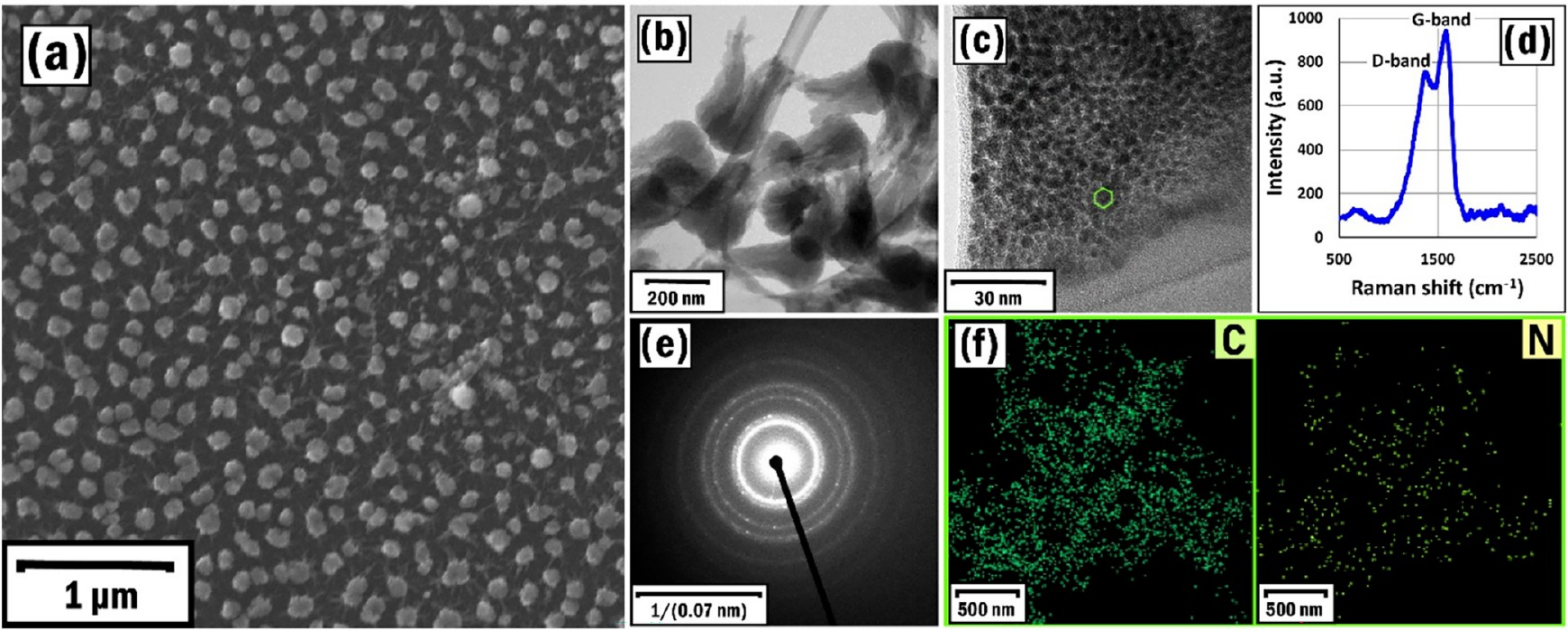
SEM image (a), low (b) and high (c) resolution TEM images, Raman spectra (d), SAED pattern (e), and EDX elemental mapping analysis of C and N elements (f) of the SP1 sample.

C–N bonds resulting from the interactions of nitrogen-assisted plasma play a critical role in the formation of the hexagonal structure and the growth of carbon nanotubes, which has been proven in various works.^22–24^ The atomic-level interaction of N (the substitution of N for C) in carbon layers lowered the energy barrier to crate pentagon structures as fullerene-like phases. Here, the degree of curvature of fullerene surfaces was correlated with the nitrogen content. Thus, the formation of CN_*x*_ nuclei on the heated substrate surface acted as the “seeds” for the free growth of the CNTs as a thermodynamically favorable precondition. Replacing nitrogen with argon has eliminated these conditions, as argon is particularly inert and devoid of the ability to form chemical bonds.^60–62^ Herein, characterization studies have also been conducted on the SP2 sample grown in 100% argon-assisted plasma without the presence of C–N bonds. In this case, rather nanoparticles with sizes of 20–30 nm were present in the SEM images of the film (Figure 4a). The smaller particles (<5 nm) were initially broken off from the graphite target and coalesced under the stable temperature conditions (400 °C) on the substrate.^63^ Although the distribution of carbon and other elements (Si, N, O, Ni) was quite homogeneous (Figure 4b–f), no favorable conditions were created for the free growth of nanotubes. The source of Si was probably the silicon substrate, while the presence of oxygen and nitrogen was due to the adsorption of atmospheric species on the surface. The EDX spectrum also contained nickel peaks (Figure 4g) reflecting the presence of nickel catalytic particles. In all measurements, the EDX analysis (Quantax 200 with an XFlash 6|10 detector (Bruker)) was carried out with a resolution of 127 eV and 15 kV of accelerating voltage in the SEM setup, and the elemental mapping analysis was conducted (Oxford Instruments) in scanning mode with a spot size of 1 nm in the TEM setup.

**Figure 4:**
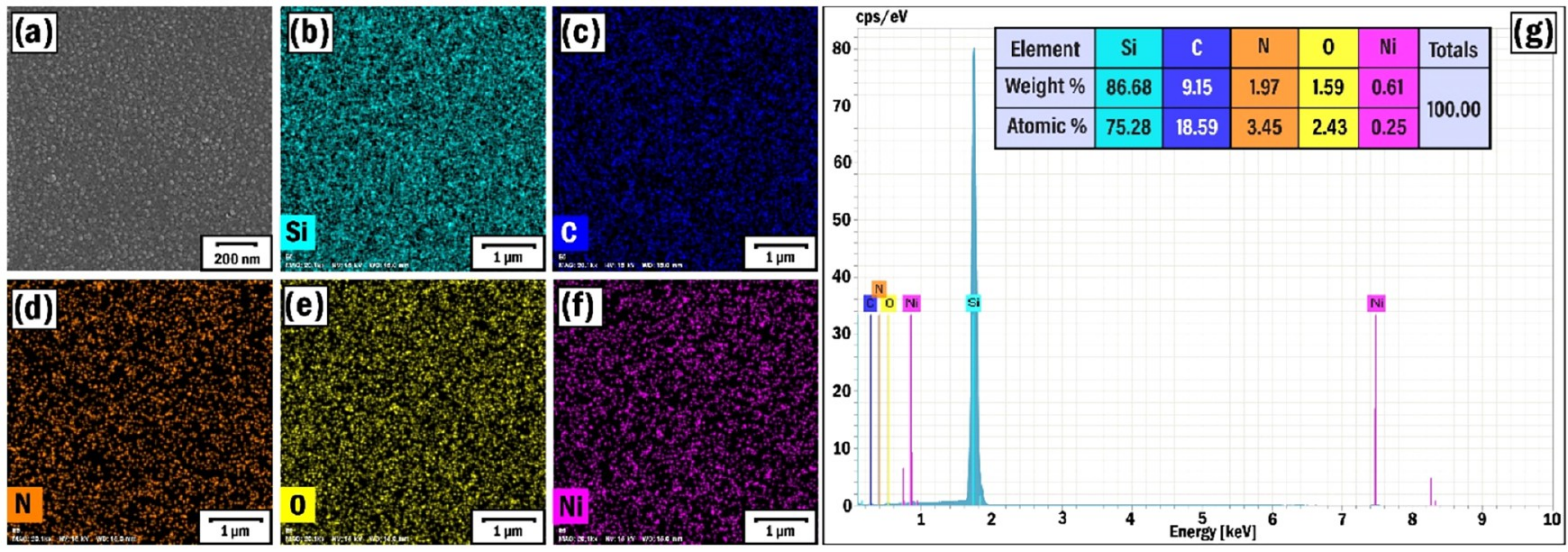
SEM (a) image, EDX elemental mapping analysis of Si (b), C (c), N (d), O (e), and Ni (f) elements, and EDX spectrum (g) of the SP2 sample.

An experiment to grow nanotubes in the presence of oxygen-assisted plasma was also carried out using the same sequence and the same preconditions (sample SP3). In this case, absolutely no nanotubes were detected, and a thick film consisting of nanoparticles (size 40–60 nm in diameter) was only formed (Figure 5a). The EDX spectrum of the film contains peaks of silicon, carbon, nitrogen, and oxygen with a clear absence of nickel (Figure 5b–e). The oxygen plasma intensively contributes to the sputtering process, which leads to the formation of larger grains, and due to the relatively low concentration of nickel catalyst particles in this grain network, they were not expressed in the spectrum. Thus, the application of oxygen plasma did not contribute to the growth of nanotubes but only slightly increased the concentration of oxygen species in the film (Figure 5f).

**Figure 5:**
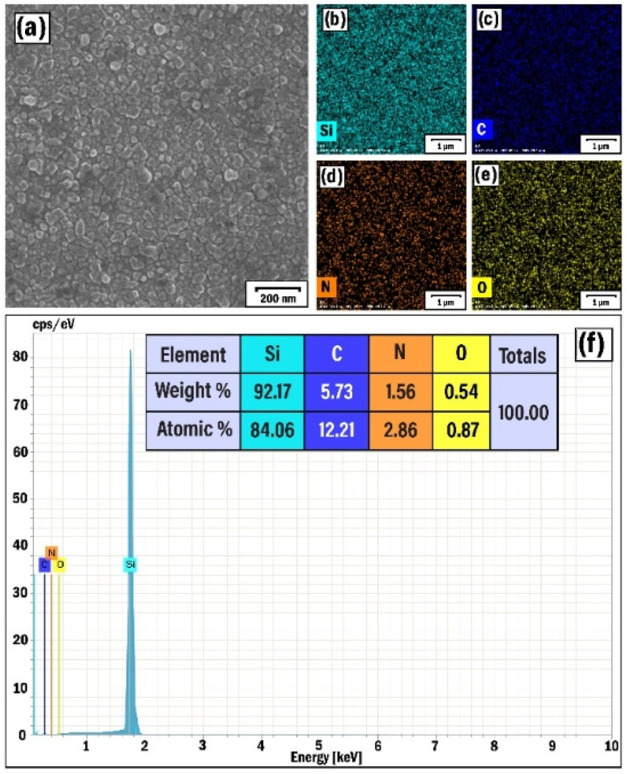
SEM (a) image, EDX elemental mapping analysis of Si (b), C (c), N (d), and O (e), elements, and EDX spectrum (f) of the SP3 sample.

It is known that the size of the particles detached from the target during magnetron sputtering is highly dependent on the power of the generator.^64,65^ One of the prerequisites for the growth of CNTs is that the nanoparticles detached from the target are small enough, even down to the size of an atom. This facilitates easier carbon adsorption around the catalytic nanoparticles and the formation of hexagonal crystal seeds.^45,66,67^ In the technological flow of the grown nanotubes, the generator power was in the range of 100–120 W,^39,42^ while at lower values, there was almost no CNT growth progress. Thus, we increased the generator power (to 140 W) to understand the real impact of this parameter on nanotube formation (sample SP4). In this case, the SEM image of the film clearly showed the growth of daisy-like nanostructures (Figure 6a), which consisted of an assembly of numerous nanobelts. The homogeneous distribution of the plasma medium and catalytic particles also led to the homogeneity of the elements (Si, C, N, and O) present here (Figure 6b–e).

**Figure 6:**
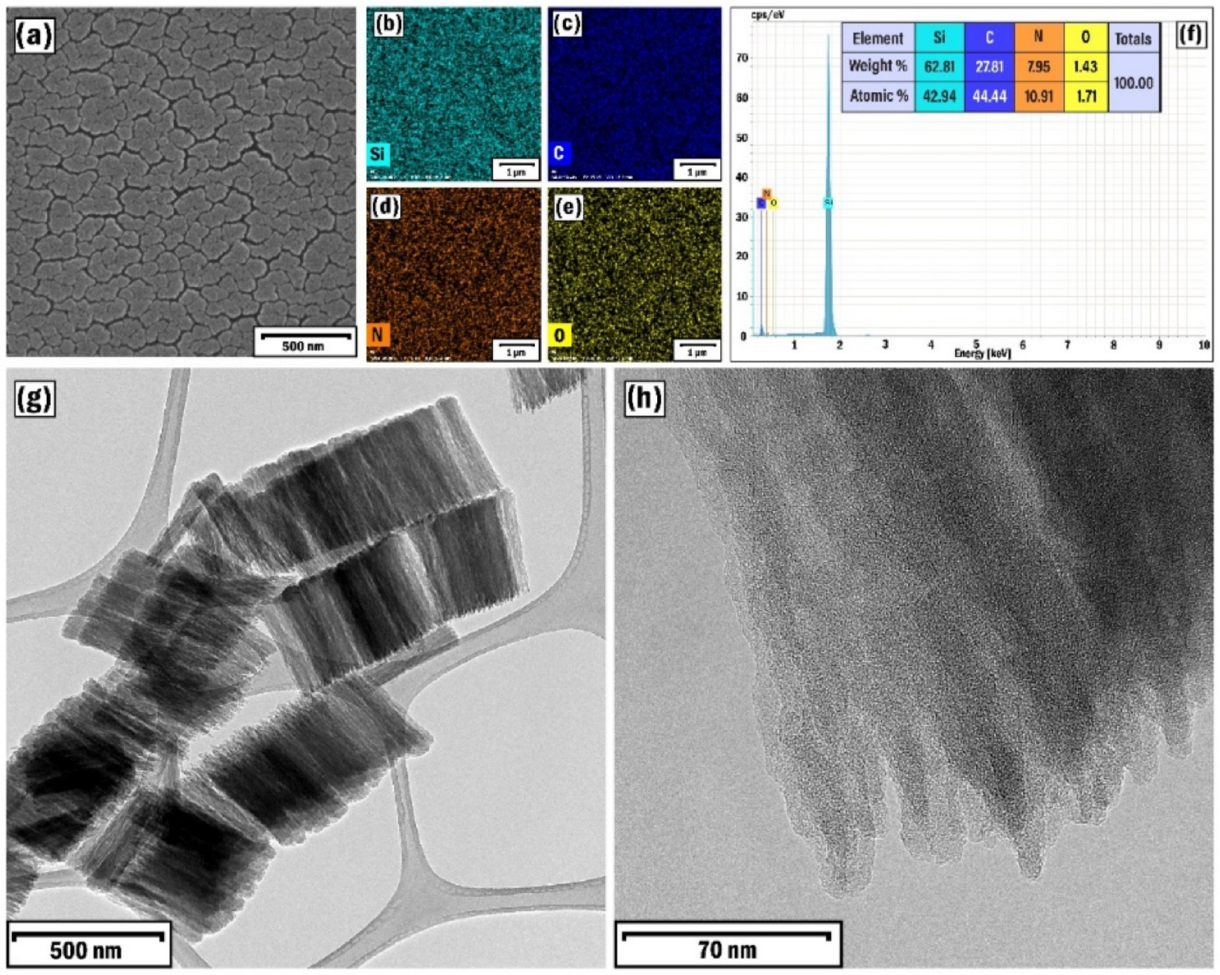
SEM (a) image, EDX elemental mapping analysis of Si (b), C (c), N (d), and O (e) elements, EDX spectrum (f), and low (g) and high (h) resolution TEM images of the SP4 sample.

The larger graphite nanoparticles detached from the target were able to form nanotubes with a larger diameter, but the growth process was accompanied by their merging. This likely prevented the complete formation of individual nanotubes, resulting in bamboo-like structures. The crystal properties of the resulting nanostructures were further elucidated using the TEM technique (Jeol 2200 FS with 200 kV of accelerating voltage). Here, the multilayered structure of the resulting nanobelts with lengths of 400–500 nm was clearly visible, representing a disordered crystalline arrangement (Figure 6g,h). At higher sputtering power, longer nanostructures were obtained than those appearing in the SP1 sample (the CNTs had 150–200 nm in length). The absence of the typical hexagonal structure leads to an increase in the rate of the deposition process.

An essential prerequisite for the growth of nanotubes is the presence of catalytic particles, around which the process of building crystallized nanotubes is formed.^68,69^ In this method, catalytic nanoparticles are formed in a very short sputtering duration, due to which a complete solid film is not formed but rather individual metal islands are obtained. By the modulation of the deposition time of catalytic particles, both their size and distribution can be controlled. The DC deposition of Ni for 20 min in the case of the SP1 sample resulted in their homogeneous distribution and obvious growth of CNTs. In an attempt to justify this growing mode, we reduced the deposition time of the catalyst nanoparticles to 10 s (SP5 sample). In this case, the growth of nanotubes was not observed, but the presence of small graphite nanograins was visible (Figure 7a). Here, the highly homogeneous distribution of individual elements (Si, C, N, and O), which was expressed in their elemental mapping images, was not observed (Figure 7b–f). The shorter nickel deposition time resulted in a lower distribution density and smaller size of the catalyst nanoparticles. Such small particle sizes are not conducive to the free growth of nanotubes, because catalyst particles smaller than a certain critical size particularly block the nanotube growth process according to the theory of the VLS.^45,70^

**Figure 7:**
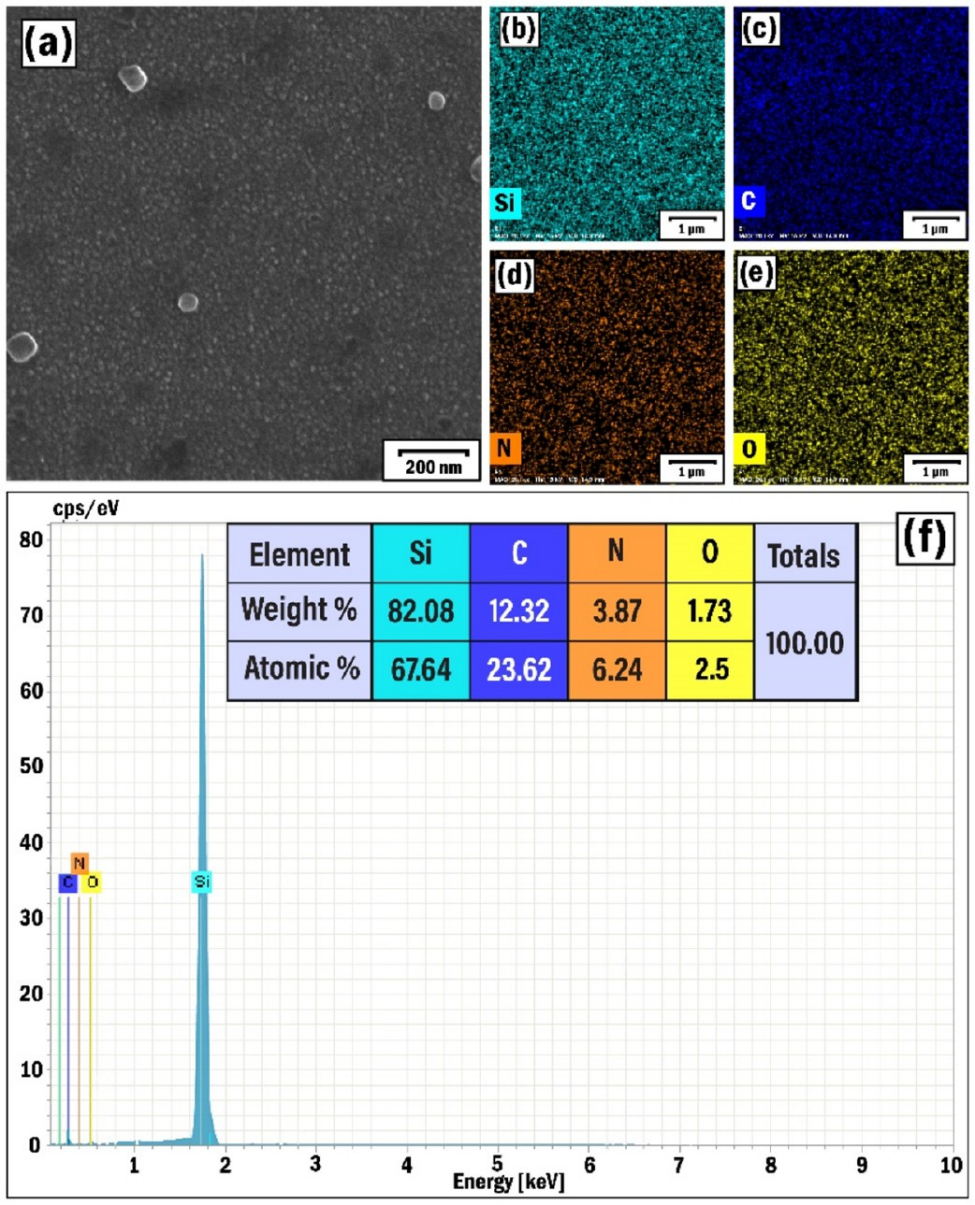
SEM (a) image, EDX elemental mapping analysis of Si (b), C (c), N (d), and O (e) elements, and EDX spectrum (f) of the SP5 sample.

To observe the reverse process, we also increased the duration of the deposition time of the catalyst particles to 30 s, while keeping the other related parameters constant (sample S6). The increasing of deposition time of nickel particles leads to the formation of denser distributed and larger nanoparticles on the substrate. This appears to have led to the barely noticeable growth of mushroom-like nanoclusters and the concomitant growth of longer nanobelts (Figure 8a). It is believed that the larger size of the catalytic nanoparticles tended to grow larger diameter nanotubes, which was inhibited by the diffusion restriction for Ni solid catalyst nanoparticles.^71^ The EDX elemental mapping images of the film containing elements (Si, C, N, O, and Ni) were well expressed, where the Ni peak appeared again (Figure 8b–g). Increasing the duration of nickel sputtering resulted in a greater amount of this material on the substrate, reflected in the EDX spectrum. The crystal structure of the resulting nanobelts was observed in their TEM images, where they more closely resembled quasi-amorphous or polycrystalline graphite formations (Figure 8h,i).

**Figure 8:**
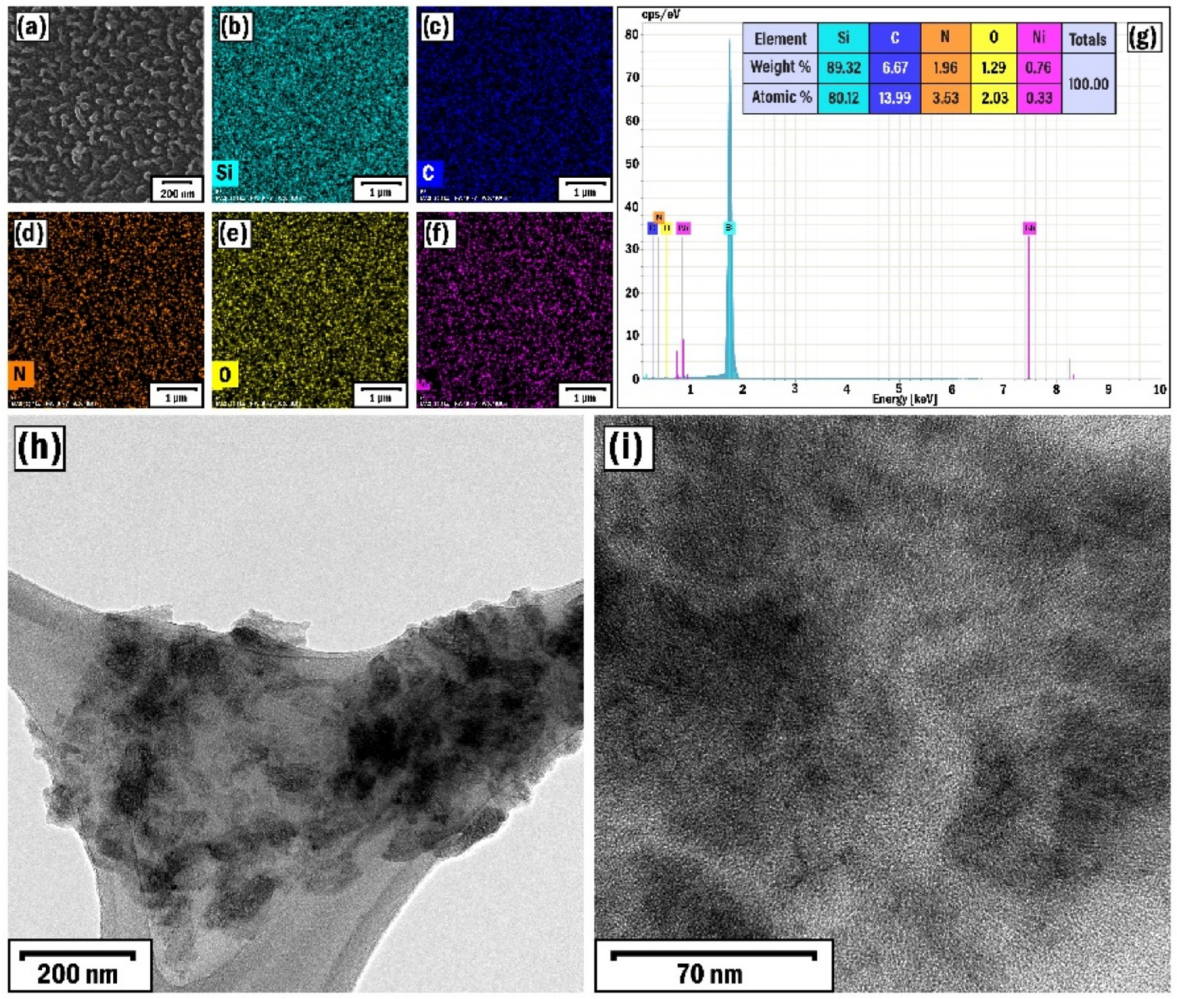
SEM image (a), EDX elemental mapping analysis of Si (b), C (c), N (d), O (e), and Ni (f) elements, EDX spectrum (g), and low (h) and high (i) resolution TEM images of the SP6 sample.

In the CNTs growth process, the most preferred versions are the use of transition metals as catalytic centers, such as Fe, Ni, and Co, the effectiveness of which has been confirmed in both PVD and CVD technologies.^72^ Latterly, noble (Ag, Au) and post-transitional metals (Pb, In) were also used as catalyst materials.^73^ For CNT growth, the use of chromium nanoparticles is rare; therefore, it was intended to conduct an experimental test for it. Keeping all technological parameters for the CNT growth process similar to those of previous experiments, we used a chromium target instead of a nickel one (sample SP7). No nanotube growth was observed in this case, but a dried peat-like surface was obtained (Figure 9a) containing Si, C, N, and O elements (Figure 9b–f). TEM images of the cross-section of the film revealed their nearly cylindrical structure with a diameter of 180–210 nm and a length of 200–300 nm (Figure 9g). The high-resolution TEM image was quite close to the polycrystalline lattice of the CNT-based structures,^42,74^ but any hexagonal trace of crystal orientation was not observed (Figure 9h).

**Figure 9:**
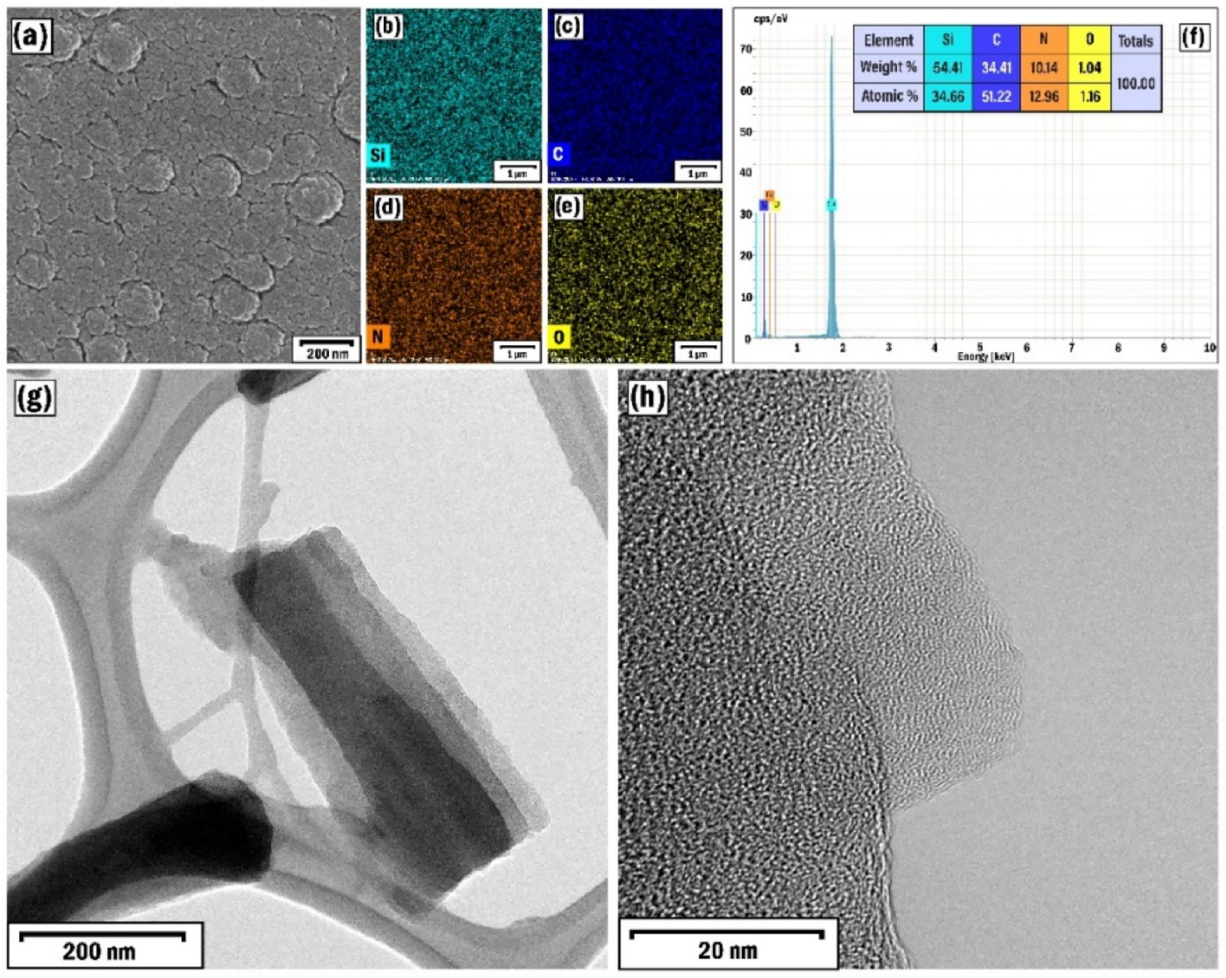
SEM image (a), EDX elemental mapping analysis of Si (b), C (c), N (d), and O (e) elements, EDX spectrum (f), and low (g) and high (h) resolution TEM images of the SP7 sample.

### Gas Sensing Studies

We manufactured a flexible sensor based on the composite of grown CNTs (sample SP1) with the Fe_2_O_3_:ZnO nanoparticles and investigated its sensitivity to PGV.^42^ The fabricated sensor showed sensitivity starting from room temperature, where the sensor speed was quite low, and it did not fully recover to the baseline resistance (Figure 10). The appearance of the real-time response curves and their values improved at higher temperatures. At the selected operating temperature of 150 °C, the sensor responded to 1.5 ppm of PGV under ultraviolet (UV) irradiation, tending to saturate the response at the higher concentrations. Here, the UV radiation generated free charge carriers in the semiconductor lattice, which reduced the semiconductor resistance and simultaneously participated in the chemisorption processes. This led to a decrease in the operating temperature of the sensor and an increase in its speed.^75^

**Figure 10:**
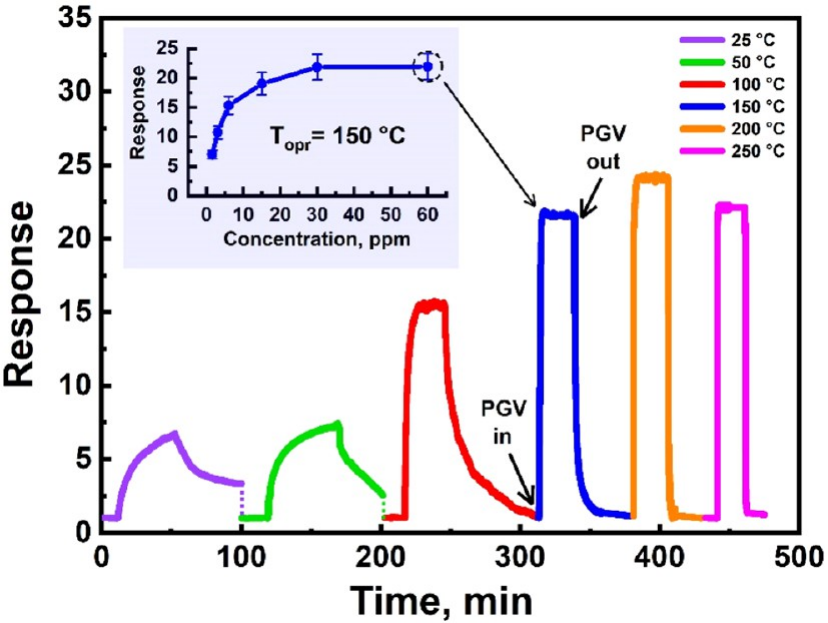
Real-time response curves of the sensor at different operating temperatures with UV irradiation at 60 ppm PGV and PGV response vs concentration at 150 °C.

The Fe_2_O_3_:ZnO/CNTs sensor was also studied by impedance spectroscopy to identify its high-frequency characteristics. Initially, the sensor’s Nyquist curves were derived in air and at a concentration of 60 ppm PGV, which had a clear semicircular shape (Figure 11a). Under the influence of PGV, the pronounced deviation of the Nyquist curves was evident as an indicator of the presence of sensitivity expressed by the impedance method. The studies were conducted with a 100 mV sinusoidal amplitude signal in a fairly wide frequency range (1 Hz–1 MHz) where the fitting of the equivalent electrical circuit with the known experimental data^76,77^ was performed, representing a capacitive component of the sensor. Thus, the equivalent electrical circuit consisting of *R*_s_ (serial resistance), *R* (resistance), and CPE (constant phase element) elements was selected (Figure 11b). It was assumed that the capacitance component was mainly due to the polycrystalline grain structure of the sensor, and it was not affected by the influence of the target gas. This scheme was in fairly good agreement with the fitting, while at the same time, the values of *n*_1_ ≈ 0.969 and *n*_2_ ≈ 0.944 representing the CPE element in air and in the presence of PGV, respectively, indicated the behavior of the CPE element as having a more capacitive nature. PGV affects the upper layer of the film surface, as a result of which the change in sheet resistance was expressed representing the value of *R*. Thus, the main contribution to the sensor response was made by the physicochemical processes occurring mainly on the top surface of the semiconductor film.^57,76,78^

**Figure 11:**
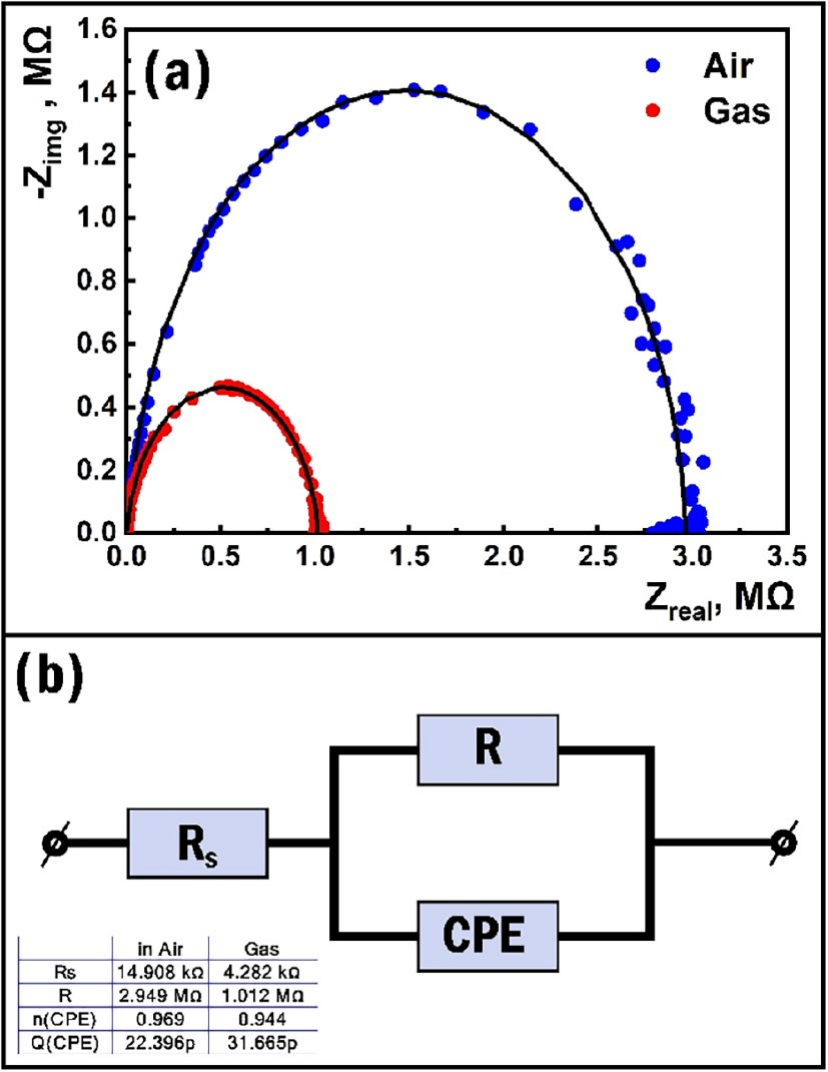
Nyquist curves of the sensor in air and in the presence of 60 ppm of PGV at 150 °C (a) and the equivalent electrical circuit of the sensor (b).

There are many ways to improve the selectivity of chemoresistive sensors, the correct choice of which is determined by the structural features of the sensor and the type of target gas.^79,80^ A rather preferred method for improving the selectivity and alternative use of the sensor is temperature modulation, which involves using the same structure as the gas-sensing element but at a different operating temperature, capable of sensing another kind of target gas.^81^ Thus, the Fe_2_O_3_:ZnO/CNTs sensor’s response to the HPV was tested in the operating temperature range of 25–150 °C, where the maximum response value (27) was observed at 50 °C (Figure 12a). At this temperature, the sensor demonstrated considerable high selectivity (Figure 12b), presenting a significantly higher response value (*S* = 27, *n* = 6 ppm) for HPV than for PGV (*S* = 7, *n* = 60 ppm), acetone (*S* = 1, *n* = 60 ppm), ethanol (*S* = 1, *n* = 60 ppm), toluene (*S* = 1, *n* = 60 ppm), water vapor (*S* = 1, *n* = 60 ppm), dimethylformamide (DMF) (*S* = 1, *n* = 60 ppm), and ammonia (*S* = 1, *n* = 60 ppm). This allowed us to use the Fe_2_O_3_:ZnO/CNTs sensor at 50 °C as a low-concentration HPV detection system, demonstrating sensitivity in the HPV concentration range of 0.5–25 ppm. The sensor was capable of sensing extremely low concentrations of HPV (0.5 ppm), where the resistance changed more than 3 times. In the case of different HPV concentrations, the sensor exhibited quite reproducible time-dependent response curves while maintaining the baseline resistance values in the absence of target gas (Figure 12c). Besides, at lower concentrations (0.5–6 ppm), the sensor exhibited a pronounced linear behavior and reached apparent saturation above 6 ppm (Figure 12d). This allows us to propose the effective use of the sensor for detecting particularly low HPV concentrations.

**Figure 12:**
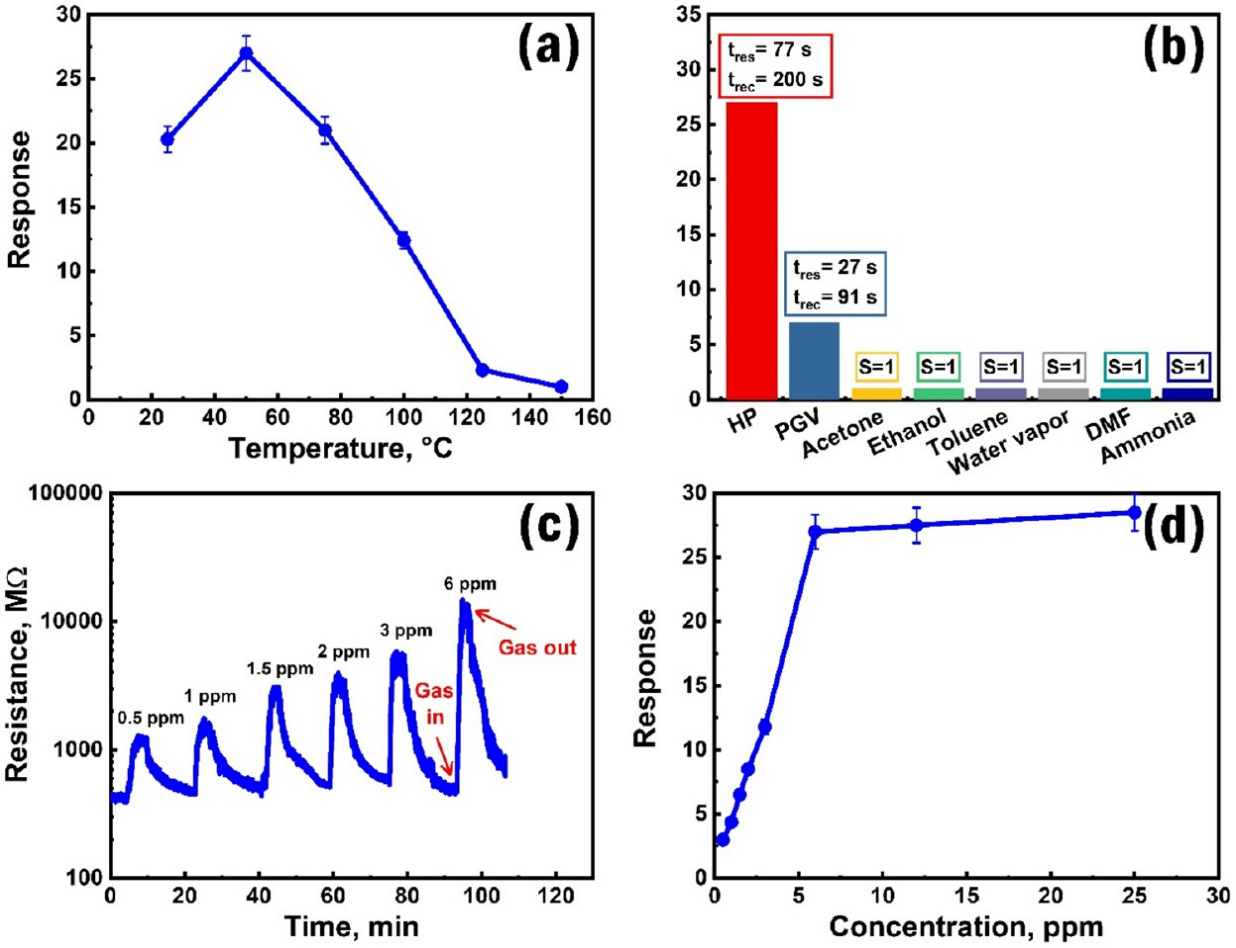
Dependence of the HPV response on the operating temperature in the range of 25–150 °C at 6 ppm of HPV concentration (a), selectivity of the sensor for HPV at 50 °C (b), HPV responses toward different concentrations at 50 °C (c), dependence of the sensor’s response on HPV concentration at 50 °C (d).

The sensor’s performance indicators included response and recovery times, which were attributed to 77 and 200 s, respectively (Figure 13a). Considering rather the low operating temperature (50 °C), these speed indicators can be considered promising. Furthermore, the repeatability of the sensor response was verified in six different measurements while maintaining the same experimental conditions. The response values recorded in individual measurements and their time-dependent curves were almost identical (Figure 13b), indicating a high stability of the sensor response. Besides, the long-term stability of the sensor response was measured over 15 consecutive days, and no significant deviations in the response from the initial value were observed (Figure 13c). As a flexible sensor, the stability of its parameters, depending on the bending number, is extremely important. Thus, the sensor response was measured after 0–1000 bending attempts, which did not significantly distort the stability of the initial response value (Figure 13d). Besides, we have actually prepared sensors based on pristine CNTs and Fe_2_O_3_:ZnO materials, both of which showed extremely poor gas-sensing results confirming the synergistic effect of the hybrid nanocomposite (CNTs/Fe_2_O_3_:ZnO).

**Figure 13:**
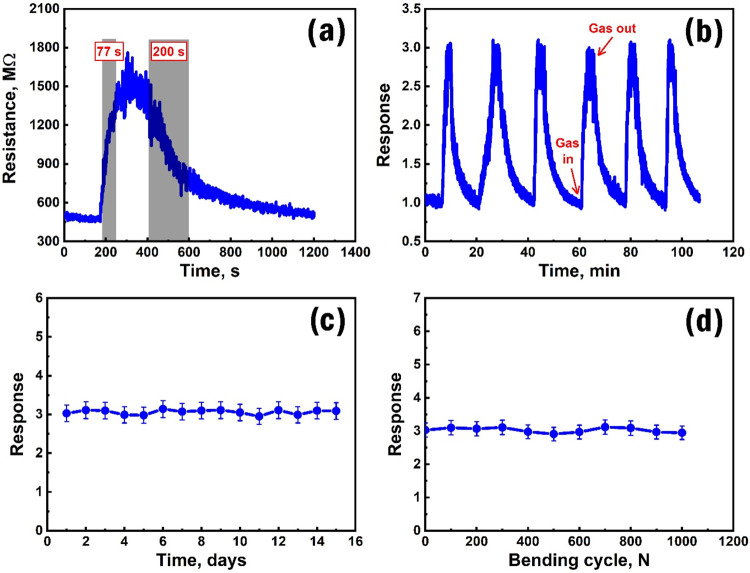
Change in the dynamic response of the sensor reflecting the response and recovery times at 0.5 ppm of HPV (a), repeatability tests of the sensor to 0.5 ppm of HPV at 50 °C (b), long-term stability of the sensor (c), and stability of the response to multiple bending of the flexible sensor for 0.5 ppm of HPV (d).

To justify the relevance and competitiveness of the fabricated sensor, its parameters were compared with those of other HPV sensors available in the literature. The sensitive materials of the various sensors and their response at the corresponding operating temperatures, presented in Table 2, prove that our sensor was competitive and workable in almost all parameters.

**Table 2:** Comparison of the Gas-Sensing Characteristics of the HPV Sensors Available in the Literature with Those of Our Sensor

sensing materials	temperature	HPV concentration (ppm)	response	references
MoS_2_/RGO composites	RT	50	12 (∼373.1%)	^82^
PEDOT:PSS (poly(3,4-ethylenedioxythiophene):polystyrene sulfonate)	RT	1.9	25%	^83^
porphyrin nanofiber/SWCNTs	RT	0.1	11.25%	^84^
SWCNTs	RT	50	1.8%	^85^
MWCNTs/Fe_2_O_3_:ZnO composite	150 °C	1.5	7.3	^86^
Pt-decorated SWCNTs	RT	2.6	2.7%	^87^
ZnO<La> nanograins	220 °C	10	2	^88^
ZnO nanograins	RT	10	5%	^89^
graphene oxides (GO)	RT	17	63%	^90^
BCCF-AgO (bacterial cellulose carbon nanofiber/AgO) composite	RT	53	60%	^91^
MWCNTs/Fe_2_O_3_:ZnO composite	RT	22	179	^86^
Fe_2_O_3_:ZnO nanograins	RT	1.5	12	^92^
CNTs/Fe_2_O_3_:ZnO composite	RT	6	20	this work
CNTs/Fe2O3:ZnO composite	50 °C	0.5	3	this work

## Conclusions

To conclude, the factors affecting the free growth of CNTs by the magnetron sputtering method were studied in detail. Nitrogen-assisted plasma replacement with 100% oxygen and 100% argon plasma did not result in nanotube growth, but rather, films consisting of amorphous graphite nanoparticles were obtained. The increased value of the generator power (to 140 W) led to the formation of daisy-like nanostructures, which consisted of an assembly of numerous nanobelts in a length of 400–500 nm with a disordered crystalline arrangement. The use of a deposition time shorter (10 s) than the initial value (20 s) of Ni catalyst particle deposition also resulted in the absence of nanotube growth. In comparison, a longer duration (30 s) exhibited barely noticeable growth of mushroom-like nanoclusters and the growth rising of longer nanobelts. To reveal the effect of a material type of catalytic particle on the nanotube growth, we used a chromium target instead of a nickel target, which represented a peat-like surface with a nearly cylindrical structure with a diameter of 180–210 nm without a hexagonal trace of crystal orientation. The sensor prepared using the combination of the grown CNTs and Fe_2_O_3_:ZnO material showed enhanced PGV sensing results, demonstrating sensitivity even at room temperature. In the research of the impedance method of the fabricated flexible PGV sensor based on the Fe_2_O_3_:ZnO/CNTs composite, the equivalent electrical circuit consisting of *R*_s_ (serial resistance), *R* (resistance), and *C* (capacitance) elements was selected. The same structure was also successfully used in the detection of HPV at 50 °C, where the sensor responded even to a parts per billion level of HPV (500 ppb). The short response (77 s) and recovery (200 s) time, high linearity at low-concentration range (0.5–6 ppm), and high temporal and bending stability were the best prerequisites for the sensor’s future real-world application. The acquired experimental results demonstrate that the growth of CNTs with optimized parameters and their application in the production of HPV and PGV sensors may open prospects for the production of commercially viable gas detectors in the future.
